# Exposition au mercure et état de santé des médecins dentistes de deux régions du centre du Maroc: enquête transversale descriptive

**DOI:** 10.11604/pamj.2020.36.110.19623

**Published:** 2020-06-19

**Authors:** Nourdine Attiya, Rkia Fattahi, Ahmed El-Haidani, Nadia Lahrach, Mohamed-Yassine Amarouch, Younes Filali-Zegzouti

**Affiliations:** 1 Laboratoire Bioactifs, Santé et Environnement, Université Moulay Ismaïl, Meknès, Maroc,; 2 Institut Supérieur des Professions Infirmières et Techniques de Santé, Errachidia, Maroc,; 3 Equipe Ethnopharmacologie et Pharmacognosie, Faculté des Sciences et Technique Errachidia, Université Moulay Ismaïl, Maroc,; 4 Laboratoire Ressources Naturelles et Environnement, Faculté Polydisciplinaire de Taza, Université Sidi Mohamed Ben Abdellah de Fès, Fès, Maroc

**Keywords:** Mercure, amalgame dentaire, exposition professionnelle, médecins dentistes, enquête transversale, Maroc, Mercury, dental amalgam, dentists, occupational exposure, cross-sectional survey, Morocco

## Abstract

**Introduction:**

l´exposition au mercure est un problème de santé publique mondialement reconnu. Si les intoxications aiguës ou subaiguës par ce métal font l´objet d´un consensus diagnostique, l´impact d´une exposition chronique à de faibles doses est encore sujet à débat. En dentisterie, le mercure est utilisé pour la réalisation des amalgames dentaires. De ce fait, les professionnels de la médecine dentaire et spécialement des médecins dentistes représentent une population exposée à ce facteur de risque. Dans ce contexte, une enquête épidémiologique descriptive, transversale et exhaustive a été réalisée afin d´évaluer cette exposition chez les médecins dentistes dans deux régions du centre du Maroc. Parallèlement, leur état de santé globale a été relevé afin de juger de la pertinence d´une enquête étiologique ultérieure pour étudier l´impact probable de l´exposition à ce facteur de risque.

**Méthodes:**

la présente étude a été réalisée par le biais d´un questionnaire auto-renseigné par les participants. L´exposition professionnelle des médecins dentistes a été évaluée en déterminant la fréquence d´utilisation de l´amalgame dentaire. Les différentes sources de mercure qui modulent la pollution de fond ou qui provoquent des pics d´exposition et qui sont en rapport avec l´environnement ou les habitudes du travail ont aussi été étudiés. Les sources d´exposition non professionnelle prises en compte sont le tabagisme, la vaccination, la consommation de poisson et la présence des amalgames dentaire en bouche. Enfin, l´état de santé des praticiens recrutés dans l´étude a été sondé globalement en se basant sur leurs déclarations.

**Résultats:**

cent quatre-vingt douze (192) médecins dentistes ont été recrutés dans cette étude. Parmi lesquels, 76,04% déclarent utiliser l´amalgame dans leur pratique avec une fréquence variable. De plus, plusieurs sources de surexposition en rapport avec les conditions ou les habitudes du travail ont été mises en évidence. La principale source d´exposition non professionnelle dans notre population est en rapport avec la présence des amalgames dentaires en bouche (63,45%). Les plaintes sanitaires exprimées par les participants concernaient en chef de file les problèmes neuropsychologiques et ce chez 46,35% de la population. En prenant en compte la neurotoxicité reconnue du mercure, une étude étiologique s´avère parfaitement justifiable dans notre contexte.

**Conclusion:**

l´exposition au mercure chez les médecins dentistes des régions étudiées est réelle. Des mesures de prévention sont à promouvoir. La réalisation d´une recherche étiologique pour juger l´impact de cette exposition est parfaitement indiquée.

## Abstract (en)

**Introduction:**

mercury exposure is recognized as a worldwide public health concern. However, the effect of long-term exposure to low-doses of this heavy metal is still subject to debate. Due to the use of mercury in dental amalgam, dental health care professionals are chronically exposed to low-doses of this metal. In this context, we have conducted a descriptive cross-sectional survey among liberal dentists in two regions of the center of Morocco. In parallel, the global health status of participants was investigated to assess the relevance of a subsequent etiological survey.

**Methods:**

data were collected through a self-reported questionnaire. Occupational exposure of dentists to mercury was evaluated based on their use of dental amalgam. Moreover, the other common factors increasing the background pollution or inducing exposure peaks were also investigated. On the other hand, smoking, vaccination, fish consumption, and the number of dental amalgam in mouth were considered as non-occupational exposure sources. Finally, the self-reported global health problems of participants were collected.

**Results:**

192 dentists were included in the present study. Seventy-six percent (76.04%) of them declared using dental amalgam in their practice. Moreover, the presence of dental amalgam in mouth was identified as the main non-occupational source of exposure to mercury (63.45% of participants). Finally, most of participants (46.35%) have expressed neuropsychological complaints.

**Conclusion:**

altogether, our results revealed a real mercury exposure in the studied population. Thus, effective preventive measures should be promoted to minimize the mercury exposure in dental offices. Moreover, an etiological study will be of great interest to reveal the impact of mercury exposure in this population.

## Introduction

La toxicité du mercure, de Pline l´ancien au I^e^ siècle de notre ère jusqu´à la convention de Minamata en 2013, n´est plus à démontrer. Cependant, si les intoxications aiguës ou subaiguës au mercure font l´objet d´un consensus diagnostique, les effets d´une exposition chronique à de faibles doses sont encore sujets à débat [1, 2]. Le mercure est un métal ubiquitaire présent sous forme liquide à température ambiante. Il se combine facilement avec d´autres molécules telles que les métaux, les molécules inorganiques ou organiques. En odontologie conservatrice, le mercure est utilisé sous sa forme élémentaire pour la réalisation des amalgames dentaires. En effet, du fait de ses propriétés mécaniques et de son coût raisonnable, l´amalgame dentaire reste le matériau de choix pour obturer les cavités carieuses nettoyées, notamment pour les dents postérieures [3]. Dans ce contexte, les médecins dentistes représentent une population exposée au risque d´intoxication chronique au mercure. En effet, ce dernier s´accumule progressivement dans l´organisme grâce aux liaisons stables qu´il crée avec les groupements thiols des protéines [4]. En plus de cette exposition professionnelle, les médecins dentistes sont sujets aux autres formes d´exposition qui concernent la population générale dont les principales sources sont les obturations dentaires à l´amalgame [2], le méthylmercure présent essentiellement dans les produits de mer [5], le thiomersal contenu dans certains vaccins [6] et le tabagisme [7]. L´ensemble de ces expositions participe à la charge corporelle au mercure responsable des troubles morbides surtout si les seuils de sécurité sont dépassés ou si les mécanismes de détoxification naturelle font défaut notamment en cas de susceptibilité génétique [8]. L´exposition professionnelle au mercure chez les médecins dentistes est un sujet qui a été largement étudié dans plusieurs pays mais très peu au Maroc. En effet, seules trois études se rapportant à la gestion des déchets des amalgames dentaires ont été répertoriées [9-11]. L´absence de données concernant sur ce sujet au Maroc, nous a mené à conduire une enquête épidémiologique dans deux régions du centre de ce pays; Fès-Boulmane et Meknès-Tafilalet. Le but principal étant d´évaluer l´exposition au mercure chez les médecins dentistes de ces régions. Dans ce sens, une analyse des différentes sources d´exposition et des facteurs l´influençant a été réalisée. De plus, les problèmes de santé générale chez la population d´étude ont été relevés pour juger de la pertinence d´une recherche étiologique ultérieure avec le mercure comme facteur de risque.

## Méthodes

**Zone d´étude:** afin d´avoir un aperçu sur la problématique de l´exposition au mercure chez les médecins dentistes au Maroc, on a opté pour la réalisation d´une enquête épidémiologique transversale et exhaustive dans deux régions du centre du pays qui sont mutuellement limitrophes mais socialement et économiquement contrastées [12]. La population source est constituée de l´ensemble des médecins dentistes exerçant à titre libérale dans la région de Fès-Boulmane et dans la région de Meknès-Tafilalet, le secteur public étant minoritaire dans l´exercice de la profession au Maroc [13]. Selon les données du ministère de la santé publiées en 2015, un effectif total de 201 médecins dentistes exerce dans la région de Fès-Boulmane contre 141 dans celle de Meknès-Tafilalet (données groupées sans localisation précise) [13]. D´autre part, il est à noter qu´après le nouveau découpage territorial au Maroc, les noms de ces deux régions sont devenus, région Fès-Meknès et Drâa-Tafilalet, respectivement.

**Recrutement des participants:** la présente enquête s´est voulue exhaustive en ciblant l´ensemble des cabinets dentaires recensés dans les pages jaunes marocaines. Les adresses de 160 médecins dentistes ont pu être obtenues pour la région de Fès-Boulmane et 111 pour la région de Meknès-Tafilalet. Après réalisation de l´enquête, un total de 192 praticiens a pu être recruté (90 médecins pour la région de Meknès-Tafilalet et 102 pour la région de Fès-Boulmane). Les participants ont été inclus dans l´étude après leurs consentements libres et éclairés.

**Déroulement de l´étude:** cette étude s´est déroulée sur une période de trois mois, du début du mois de février à la fin du mois d´avril 2016. Elle consistait en la distribution d´un questionnaire avec un appel à participation où sont expliqués la problématique et les objectifs de l´étude, son cadre purement académique de recherche scientifique et son caractère basé sur l´anonymat et le consentement libre et éclairé des participants. Les questionnaires étant auto-renseignés, une deuxième visite était réalisée pour leur collecte.

**Le questionnaire:** l´enquête a été réalisée par le biais d´un questionnaire auto-renseigné par les participants. Ce questionnaire peut être scindé schématiquement en quatre parties distinctes: 1) la première concerne l´état civil et les renseignements personnels des participants; 2) la deuxième est dédiée à l´exposition non professionnelle au mercure: on a tenu compte de quatre sources essentielles qui sont le statut tabagique et vaccinal des sujets, la fréquence de la consommation de poisson en spécifiant les espèces couramment consommées et le nombre des obturations dentaires à l´amalgame intra-orales. On a également étudié les facteurs de « surexposition » qui sont associés à cette dernière variable, à savoir, la consommation de chewing-gum, la présence de bruxisme ou de respiration buccale et la présence d´autres dispositifs métalliques intra-buccaux. Ces facteurs augmentent la libération du mercure contenu dans les amalgames durcis [14, 15]; 3) la troisième partie est consacrée à l´exposition professionnelle où l´on a spécifié la fréquence d´utilisation de l´amalgame dentaire comme matériau d´obturation dans l´exercice quotidien ainsi que les différents facteurs qui influencent l´intensité de cette exposition et qui sont en rapport avec l´environnement du travail ou avec les habitudes personnelles de préparation ou de manipulation de l´amalgame ainsi que les moyens d´hygiène et de protection individuelle [4, 15]; 4) la dernière partie était focalisée sur l´état de santé générale des participants en insistant sur les différents systèmes dont l´atteinte pourrait être en relation avec une intoxication chronique au mercure. Le but de cette dernière partie est d´évaluer les besoins sanitaires exprimés par la population étudiée et de préparer l´enquête étiologique ultérieure.

**Gestion des données:** les données ont été saisies, validées et « nettoyées » avec le logiciel EPI7. L´exploitation statistique a été réalisée avec le logiciel R version 3.5.2. Les variables quantitatives ont été exprimées en moyenne arithmétique ± écart type (SD). Les variables qualitatives ont été exprimées en répartition proportionnelle (RT). L´analyse de variance ANOVA a été adoptée pour la comparaison des moyennes. En cas de non-respect de la normalité ou de l´homoscédasticité, le test non paramétrique Kruskal-Wallis a été choisi. Enfin le test Chi^2^ a été choisi pour les variables qualitatives. Le niveau de probabilité pour lequel l´hypothèse nulle est rejetée est p < 0,05.

## Résultats

**Caractéristiques de la population étudiée:** suite à la réalisation de l´enquête, des taux de participation de 63,83% et de 56,73% ont été obtenus pour la région Meknès-Tafilalet et Fès-Boulmane, respectivement et ce en prenant comme base les données du ministère de la santé. Par la suite, les caractéristiques de la population d´étude ont été stratifiées en fonction de la région concernée (Tableau 1). Les variables considérées ont été l´âge, l´indice de masse corporelle, l´ancienneté dans l´exercice de la profession, le sexe et le milieu d´exercice (grande ville versus petite ville). Les résultats obtenus révèlent une différence significative pour les variables âge et ancienneté dans l´exercice de la profession entre les deux régions (Tableau 1). En effet, on observe une moyenne d´âge plus élevée et une plus grande ancienneté d´exercice pour la région de Fès-Boulmane en comparaison avec celle de la région de Meknès-Tafilalet. De plus, pour les deux régions, une majorité des praticiens a choisi de s´installer dans les grandes villes. En revanche, aucune différence significative n´a été observée selon la région ou le milieu d´exercice pour la variable sexe ou indice de masse corporelle. Une prédominance masculine relative est cependant notable sur l´ensemble de la population (Tableau 1).

**Tableau 1:** répartition des caractéristiques des médecins dentistes en fonction de la région

	Région Fès-Boulmane		Région Meknès-Tafilalet	
Caractéristiques	n	Moyenne ± SD	n	Moyenne ±SD
**Age a,b**	102	38,02 ± 9,33	89	34,58 ± 9,22
Ancienneté a,b	102	11,84 ± 8,25	89	8,19 ± 8,24
Indice de masse corporelle	78	25,21 ± 3,41	67	25,42 ± 3,20
**Sexe**	N	Proportion (%)	n	Proportion (%)
Femme	48	47,52	32	36,78
Homme	53	52,48	55	63,22
**Milieu d’exercice**	**N**	**Proportion (%)**	**n**	**Proportion (%)**
Grande ville	98	96,08	59	65,56
Petite ville	4	3,92	31	34,44

a: différence significative en fonction de la région b: différence significative en fonction du milieu d’exercice

**Exposition non professionnelle au mercure:** en dehors de leur exposition professionnelle, les médecins dentistes peuvent également être exposés au mercure dans leur vie quotidienne. La principale source de mercure élémentaire dans la population générale est la présence d´obturation à l´amalgame en bouche [2, 14]. La consommation fréquente de chewing-gum, la bruxomanie et la respiration buccale exacerbent le degré d´exposition [14, 15]. La présence d´autres dispositifs métalliques en bouche augmente la quantité de mercure libérée par électro-galvanisme [16]. Dans notre population, 63,45% des sujets recrutés dans l´enquête sont traités par des amalgames dentaires et les facteurs de surexposition sus-cités sont présents chez des proportions non négligeables de cette sous population (Tableau 2). L´exposition au mercure organique est surtout alimentaire par la consommation de poissons pollués [5, 17]. Les espèces prédatrices sont plus chargées en méthylmercure par bioaccumulation dans la chaine alimentaire [5]. Dans notre population, la consommation de poisson est relativement faible et se limite aux espèces de petites tailles, en l´occurrence, la sardine, le merlan et la sole. La deuxième source d´exposition au mercure organique est le thiomersal (= éthylmercure) utilisé comme conservateur dans certains vaccins [6]. Malgré le risque biologique encouru par les médecins dentistes, le taux de vaccination déclaré est de seulement 17,58% et concerne dans sa totalité l´hépatite B et la grippe. Ces deux vaccins risquent de contenir de l´éthylmercure si la formule de fabrication initiale est maintenue par les laboratoires [18]. La dernière source d´exposition prise en considération est le tabagisme puisque le tabac contient une panoplie de toxiques dont le mercure [7]. Le taux de tabagisme dans notre population est très faible (9,09%). Les résultats concernant ces différentes sources d´exposition extra-professionnelle sont résumés dans le Tableau 2.

**Tableau 2:** répartition proportionnelle des médecins dentistes en fonction des sources d’exposition non professionnelle

Source d’exposition	Modalités	Répartition proportionnelle	
		n	Proportion (%)
**Tabagisme**	Présence	17	9,09
	Absence	170	90,91
	**Total**	187	100,00
**Vaccination**	Présence	32	17,58
	Absence	150	82,42
	**Total**	182	100,00
**Consommation de poisson (nombre de repas/semaine)**	0	4	2,16
	1	83	44,86
	2	77	41,62
	3	21	11,35
	**Total**	121	100,00
**Amalgame en bouche**	Présence	122	63,54
	Absence	70	36,46
	**Total**	192	100,00
**Facteurs de surexposition chez les porteurs d’amalgames**			
**Facteur**	**Moyenne ± SD**	**Range ; EI a**	**Proportion (%)**
Nombre d'amalgame	3,10 ± 1,98	[1,10]; 3	-
Chewing-gum/jour	2,99 ± 2,53	[0,8]; 4	77,87
Présence d'autre métal	-	-	24,74
Bruxisme	-	-	56,52
Respiration buccale	-	-	15,00

a: Range: [Minimum;Maximum]; EI: Ecart interquartile

**Figure 1:**
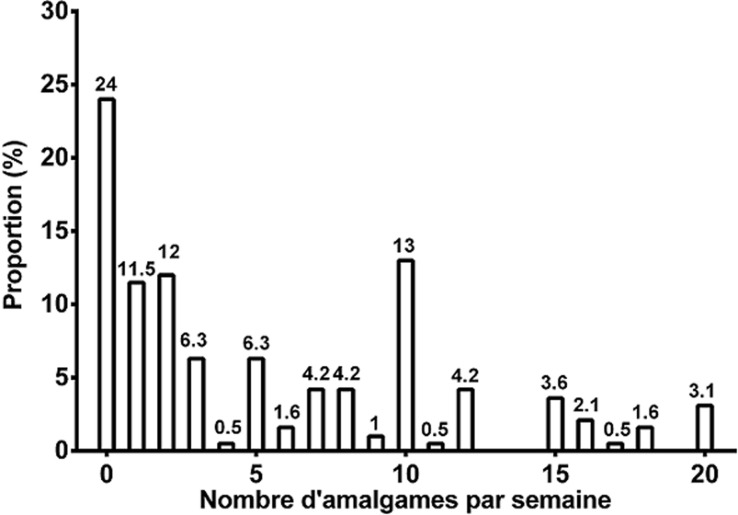
répartition proportionnelle des médecins dentistes en fonction du nombre d´obturation à l´amalgame réalisées par semaine

**Exposition professionnelle au mercure dentaire:** la principale source d´exposition professionnelle au mercure chez les médecins dentistes est l´utilisation de l´amalgame comme matériau d´obturation dentaire dans leur exercice. La fréquence de cette utilisation diffère selon les praticiens. Alors que 23,96% ont déclaré ne plus utiliser l´amalgame dentaire dans leur pratique, 76,04% continuent à en faire usage selon une fréquence variable entre praticiens. La Figure 1 met en relief cette variabilité. Si la fréquence d´utilisation de l´amalgame dentaire signe l´exposition courante, l´intensité de cette exposition est modulée par plusieurs autres facteurs en rapport avec les caractéristiques des locaux de travail et les règles d´hygiène. En effet, la pollution de fond qui présente des variations spatio-temporelles importantes est tributaire de la présence ou de l´absence de ces facteurs [4]. La nature du revêtement des sols joue un rôle important dans le maintien et l´augmentation de la pollution mercurielle de l´atmosphère ambiant. Le carrelage, du fait de son caractère usiné, présente un bon état de surface facilitant le nettoyage et retenant peu de débris sauf ponctuellement au niveau des joints mal faits, des plinthes ou en cas de fissure [4, 19]. Il constitue la majorité des sols des cabinets de notre population avec une proportion de 90,18%. La mosaïque dont la confection et le polissage sont réalisés manuellement présente un état de surface de moindre qualité que le carrelage favorisant la rétention des débris ce qui augmente *in fine* la pollution de fond. Elle constitue le sol de 9,82% des cabinets de notre population. La moquette, veritable

piège à mercure [15], n´est présente que dans un seul cabinet. Le type des sols est en relation étroite avec la méthode de leur entretien. Le lavage à l´eau, plus efficace et moins polluant est le plus utilisé avec une proportion de 86,34%. L´aspiration est utilisée pour l´entretien de 14,56% des cabinets dentaires et le balayage manuel est choisi par 4,43% de notre population. Ces deux techniques sont plus polluantes que la première [4, 15]. Le type de ventilation joue également un rôle important dans les variations de la pollution de fond. L´aération naturelle avec ouverture régulière des fenêtres est plus protectrice [4, 15]. Elle constitue le moyen utilisé dans la majorité des cabinets avec une proportion de 81,01%. Une proportion de 44,39% possède des rideaux en tissus, véritable piège à mercure et une proportion de 15,89% a eu des incidents lors de la manipulation du mercure. En relation avec les habitudes professionnelles de manipulation de l´amalgame dentaire plusieurs attitudes qui augmentent l´exposition au mercure ont été notées. 53,06% utilisent encore l´amalgame en vrac. La stérilisation est réalisée par le poupinel qui utilise la chaleur sèche chez 41,45% de notre population. Cette stérilisation est réalisée dans la salle des soins dans 36,72% des cabinets. 20,15% des praticiens travaillent sur les amalgames durcis (polissage ou dépose) sans utilisation de spray d´eau de refroidissement. Concernant le risque pour l´environnement, 78,13% jettent les déchets d´amalgame dans les poubelles à l´air libre et 71,43% utilisent le réseau des déchets ménagers pour s´en débarrasser. L´ensemble de ces résultats sont résumés dans le Tableau 3 et le Tableau 4. Les moyens de protection individuelle, bien que conçus à la base pour prévenir le risque biologique, peuvent jouer un rôle dans la protection contre le mercure [4, 15]. Dans notre population, bien que 100% des praticiens aient déclaré porter systématiquement des gants et des habits professionnels, le port du masque n´est systématique que chez 92,75% et 0,72% ont déclaré ne jamais le porter. Les chaussures professionnelles ne sont portées que par 46,35% et les bonnets par 35,94%. Dans notre enquête, on a constaté qu´aucun cabinet dentaire n´a déjà subi une décontamination contre le mercure. L´ancienneté des sols varie de 1 an à 25 ans avec une moyenne de 5,53 ans.

**Tableau 3:** répartition proportionnelle des facteurs qui influencent l’exposition au mercure dans les cabinets dentaires des régions Fès-Boulmane et Meknès-Tafilalet

Facteurs	Modalités	Répartition proportionnelle	
		Effectifs	Proportions (%)
**Nature du revêtement des sols**	Moquette	1	0,89
	Carrelage	100	90,18
	Mosaïque	11	9,82
	**Total**	111	100,00
**Type de ventilation**	Aération naturelle	128	81,01
	Climatiseur	23	14,56
	Ventilateur électrique	7	4,43
	**Total**	158	100,00
**Entretien des sols**	Lavage	139	86,34
	Aspiration	14	8,68
	Balayage	8	4,98
	**Total**	161	100,00
**Présence de rideaux en tissu**	Oui	122	74,39
	Non	42	25,61
	**Total**	164	100,00
**Type d’amalgame**	Amalgame en vrac	78	53,06
	Amalgame en capsule	69	46,94
	**Total**	147	100,00
**Mode de stérilisation**	Poupinel	63	41,45
	Autoclave	89	58,55
	**Total**	152	100,00
**Lieu de stérilisation**	Salle de soins	47	36,72
	Local à part	81	63,28
	**Total**	128	100,00
**Dépose et polissage de l’amalgame**	Avec irrigation	107	79,85
	A sec	27	20,15
	**Total**	134	100,00

**Tableau 4:** incidents de manipulation du mercure et gestion des déchets des amalgames dans les cabinets dentaires des régions Fès-Boulmane et Meknès-Tafilalet

Facteurs	Modalités	Répartition proportionnelle	
		n	Proportion (%)
**Rejet des déchets**	Récipient ouvert	100	78,13
	Récipient fermé	28	21,87
	**Total**	128	100,00
**Gestion des déchets**	Réseau municipal	35	71,43
	Réseau hospitalier	11	22,45
	Autre (non spécifié)	3	6,12
	**Total**	49	100,00
**Incidents de manipulation**	Oui	24	15,89
	Non	127	84,11
	**Total**	151	100,00

**Figure 2:**
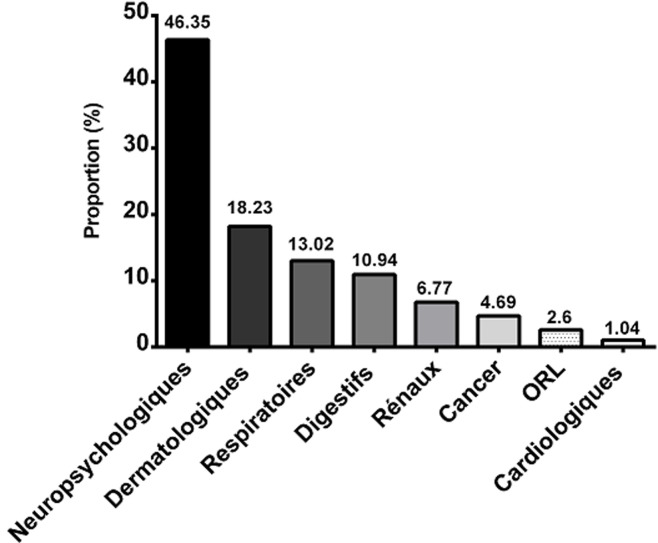
répartition proportionnelle des troubles de santé déclarés par les médecins dentistes recrutés dans l'étude

**Problèmes de santé globaux auto-déclarés:** des problèmes de santé générale ont été recherchés au niveau des systèmes les plus concernées par l´intoxication chronique au mercure et ce sans aucune démarche diagnostique. Le but étant d´évaluer les besoins de santé et guider la recherche étiologique envisagée ultérieurement. 32,50% des participants ont déclaré sentir leur état de santé se dégrader graduellement depuis le début de leur exercice. Dans notre population les problèmes neuropsychologiques sont les plus fréquents avec une proportion de 46,35%. Ils sont suivis par les problèmes dermatologiques présents dans 18,23% des cas. Les problèmes respiratoires sont évoqués par 13,02% des praticiens. Les troubles digestifs constituent la plainte de 10,94% des praticiens. Les autres troubles sont présents dans des proportions beaucoup moins importantes. La Figure 2 met en relief l´ensemble de ces résultats.

## Discussion

**Caractéristiques de la population étudiée:** afin d´investiguer les caractéristiques démographiques de la population, une stratification selon la région d´étude a été réalisée. Les résultats obtenus montrent une différence significative de l´âge et de l´ancienneté de l´exercice entre les deux régions. Cette différence peut trouver son origine dans la répartition contrastée en fonction du milieu d´exercice. En effet, cette différence est plus marquée après stratification selon le milieu d´exercice. Ce constat pourrait être lié au choix du lieu d´installation des cabinets dentaires privés. Les praticiens de l´ancienne génération pencheraient plutôt pour les grandes villes alors que celles-ci seraient devenues moins attrayantes pour la nouvelle génération. D´autre part, on note qu´à l´instar de plusieurs études réalisées dans des pays comparables au Maroc (Egypte, Turquie ou Iran) [20], notre population présente une moyenne d´âge relativement jeune et une ancienneté d´exercice comparables aux pays précités. En revanche, ces caractéristiques sont différentes dans les populations de praticiens occidentaux, où la moyenne d´âge est plus grande (Norvège, Suède, Nouvelle Zélande ou les Etats Unis) [20].

**Evaluation de l´exposition non professionnelle au mercure:** bien que l´objectif principal de notre enquête est l´évaluation de l´exposition professionnelle au mercure, la détermination des facteurs d´exposition non professionnelle est tout aussi importante car elle participe à la charge corporelle totale en mercure et constitue de ce fait un facteur de risque propre ainsi qu´une source de biais de classement dans l´étude étiologique de l´exposition professionnelle. La principale source d´exposition non professionnelle au mercure élémentaire dans la population générale est en rapport avec la présence de l´amalgame dentaire en bouche [2, 14]. En effet, il a été démontré que les amalgames dentaires ne sont pas stables mais libèrent progressivement le mercure qu´ils contiennent [16]. Dans notre population, une proportion de 63,45% de praticiens a déclaré posséder au minimum une obturation à l´amalgame et un maximum de dix amalgames, ce qui rend cette source d´exposition effective chez la majorité des participants. Dans une étude suédoise sur l´exposition au mercure de différentes origines chez les dentistes et les assistantes dentaires [14], les mercuriuries sont significativement corrélées au nombre des surfaces dentaires obturées à l´amalgame dans un modèle de régression linéaire multiple. Cette corrélation significative est retrouvée aussi bien chez les professionnelles que chez les témoins. Le taux de mercure excrété chez les témoins non exposés professionnellement varie de 0,25 à 8μg/l avec une moyenne de 2,5μg/l. Dans l´étude de Langwörth *et al*. [21], 40% environ des valeurs retrouvées chez les sujets non exposés professionnellement étaient sous le seuil de détection de sa méthode de mesure qui était de l´ordre de 2μg/l. Nilsson and Nilsson [19] ont retrouvé un taux médian dans le groupe témoin sans exposition professionnelle de 2,2μg/l pour les hommes et 3,3μg/l pour les femmes et ce pour une moyenne de 25 surfaces dentaires obturées à l´amalgame. Ces mercuriuries relativement faibles en relation avec les obturations à l´amalgame dentaire sont retrouvés dans plusieurs autres études [4]. Selon Echeverria *et al*. [22], ces mercuriuries « spontanées » sont le témoin d´une exposition récente et ne reflètent pas la charge corporelle en mercure qui est responsable de l´intoxication. Dans cette étude, les auteurs suggèrent que les mercuriuries mesurées après des tests de mobilisation du mercure par un chélateur ont une meilleure corrélation avec les signes cliniques en comparaison avec les mercuriuries « spontanées ». En effet, le dosage urinaire du mercure est un bon indicateur de l´exposition mais reflète mal le degré de l´intoxication [4]. D´autre part, selon l´Organisation Mondiale de la Santé: « le mercure a pour caractéristique une toxicité par « rétention » et la plupart du mercure pénétrant dans le corps est absorbé par les tissus solides. Le taux urinaire représente le mercure en cours d´élimination. Cependant, la question principale est de déterminer quelles quantités sont retenues dans les différents tissus du corps » [23]. Les obturations à l´amalgame, bien que ne provoquant que de faibles mercuriuries doivent être pris en compte comme source de mercure compte tenu de la chronicité de l´exposition susceptible de provoquer des signes d´intoxication [2]. La corrosion des obturations à l´amalgame responsable de la libération du mercure qu´elles contiennent est augmentée de 3 à 5 fois par l´abrasion mécanique due à la consommation de chewing-gum [14, 15]. Selon Vimy *et al*. [24], cette augmentation est 6 fois supérieure et atteint la concentration de 30μg/m^3^ en intra-oral chez les porteurs d´amalgame. Cette valeur n´est que de 0,72μg/m^3^ dans les bouches exempte d´amalgame et ce après 10 minutes de mastication d´un chewing-gum. Le bruxisme par le même mécanisme de friction générée augmente considérablement la quantité de mercure libérée [25]. La respiration buccale augmente la quantité de mercure libéré par les obturations d´amalgame [15]. La présence de ces facteurs chez des proportions importantes de notre population augmente l´exposition courante au mercure et à la longue la charge corporelle. La corrosion des amalgames est aussi électrochimique par électrogalvanisme où la salive joue le rôle d´électrolyte. Ce phénomène s´exerce à l´intérieur de la même obturation (mélange de plusieurs métaux) et entre les amalgames de marque et d´âges différents. Ce phénomène est exacerbé en présence d´autres dispositifs métalliques en bouche où l´amalgame joue le rôle d´anode corrodable car il est moins noble que les autres métaux utilisés en dentisterie [16, 26].

Presque le quart des porteurs d´amalgame dans notre population possèdent un autre métal en bouche. La deuxième source d´exposition non professionnelle étudiée est la consommation de poissons. Or, l´analyse des résultats montre que cette source est beaucoup moins importante que la première en prenant en compte le nombre de repas hebdomadaires où l´on consomme du poisson (entre 1 et 3 repas maximum) et les espèces les plus consommées (sardine, merlan et sole) qui sont de petites tailles et donc faiblement polluées par le mercure. Selon les données de l´Office National de Sécurité Sanitaire des produits Alimentaires du Maroc (ONSSA) sur la période de 2010 à 2016, la teneur moyenne en mercure dans les produits de la pêche débarqués au niveau des deux littoraux marocains est de 0,073mg/Kg, soit très en deçà des limites réglementaires en vigueur [27]. Dans l´étude de Goodrich *et al*. [17] aux Etats Unis, les professionnels de santé dentaire consommaient en moyenne 10,2 repas de poisson par mois et les taux de mercure mesurés dans le sang et les cheveux étaient très significativement supérieurs chez les consommateurs de poisson par rapport à ceux qui n´en consommait pas. Les espèces les plus consommées dans cette étude sont le thon, les crevettes, le saumon et le tilapia. Dans une étude marocaine réalisée en milieu rural [28] (exposition non professionnelle), les mercuriémies dépassaient rarement 5μg/l (3,7%) avec comme sources principales d´exposition au mercure, la consommation de poisson et de produits agricoles irrigués par des eaux usées. Les vaccins sont une source beaucoup moindre d´exposition en prenant en compte le faible taux des personnes vaccinées (17,58%). Les vaccins contre la grippe et l´hépatite B contiennent effectivement le thiomersal (= éthylmercure) à la dose de 25μg/0,5ml pour le vaccin de l´hépatite B et de 25μg/0,25ml pour celui de la grippe [6]. Bien que les études épidémiologiques réalisées par les CDC (Centers for Disease Control and Prevention) n´aient révélé aucun effet toxique du mercure contenu dans les vaccins surtout chez le nourrisson, par précaution l´Agence Européenne d´Evaluation des Médicaments a exigé des laboratoires producteurs de vaccins contenant du thiomersal, un plan d´action pour le retrait de ce conservateur de leurs formules de fabrication et ce depuis l´année 2000 [6]. A ce jour, on ne dispose pas d´information sûre sur le retrait effectif de l´éthylmercure [18] surtout au Maroc, lieu de notre enquête. Le tabagisme comme source d´exposition au mercure n´intéresse qu´une très faible proportion (9,09%) de notre population. La consommation de tabac joue un double rôle dans notre contexte, d´abord par la quantité de mercure que contient chaque cigarette consommée [7] mais aussi en augmentant la quantité de mercure inhalée en cas de manipulation des amalgames dentaires à main nue [4].

**Exposition professionnelle au mercure:** notre stratégie pour évaluer l´exposition au mercure est différente de la plupart des études réalisées dont la mesure du mercure dans les milieux biologiques et notamment dans les urines est presque toujours systématique [4,20]. En effet, si les taux urinaires sont d´une grande utilité pour évaluer l´exposition récente ou l´impact de tel ou tel facteur sur son intensité, son efficience pour évaluer la charge corporelle et par conséquent l´intoxication surtout dans le cas d´une exposition chronique à faible dose est discutable [29]. Pour cette raison, on a opté pour une approche plutôt qualitative pour évaluer l´exposition. La pierre angulaire de cette exposition est la fréquence d´utilisation des amalgames dentaires modulée par plusieurs facteurs qui influencent son intensité en ayant en arrière-plan la durée de l´exposition. Dans notre population, 23,96% des enquêtés n´utilisent plus l´amalgame dentaire, proportion légèrement inférieure à celle enregistrée en France qui est de 30% en 2011 [3]. Le nombre relativement bas des restaurations à l´amalgame réalisées par semaine reflète la perte de leur popularité par rapport aux autres matériaux chez une grande partie de notre population et ce en prenant en compte la très haute prévalence des caries dentaires au Maroc [30]. En France, l´amalgame dentaire représente moins de 25% de l´ensemble des restaurations directes réalisées en 2011 avec des prévisions de diminution de 10% pour l´année 2012 [3]. Dans l´étude de Goodrich aux Etas Unis, réalisée en 2012, seulement 15% des praticiens ont cessé d´utiliser l´amalgame dentaire et le nombre hebdomadaire chez les utilisateurs atteint les 200 amalgames manipulés (pose ou dépose) par semaine [17]. Le mercure élémentaire au contact de l´air est un toxique redoutable. Selon Caitucoli *et al*, une gouttelette de 2,5mm peut amener à vapeur saturante (400μg/m^3^) l´atmosphère d´une pièce de 50m^3^ [4]. Plusieurs études ont mesuré les taux atmosphériques du mercure dans les cabinets dentaires pour identifier les différentes sources de pollution. En 1986, Nilsson and Nilsson [19] ont trouvé des concentrations importantes dans les cabinets privés par rapport aux publics. Les concentrations les plus importantes ont été enregistrées prés du collecteur de débris d´amalgame, des poubelles, des fissures du revêtement du sol et de la table de préparation de l´amalgame. Langwörth *et al*. [21] ont démontré que les taux atmosphériques étaient fortement liés aux éventuelles pertes de mercure, aux résidus d´amalgame non récupérés ou mal conditionnés, à la nature du revêtement de sol et au type de ventilation. En effet, selon la nature des sols et la présence de rideaux, de moquettes ou de tapis, des pièges à mercure sont constitués avec relargage progressif de vapeur [15]. La ventilation en circuit fermé, la climatisation et les ventilateurs favorisent le confinement et augmentent l´exposition [4]. La stérilisation par la forte chaleur qu´elle requiert est une procédure très génératrice de vapeur de mercure si le matériel n´est pas débarrassé des débris de mercure [4, 15]. Le poupinel est théoriquement plus polluant que l´autoclave puisque qu´il utilise la chaleur sèche et sa fermeture n´est pas hermétique. La durée de l´exposition est plus importante si la stérilisation est réalisée dans la salle de soins plutôt que dans un local peu fréquenté qui lui est dédié, ce qui constitue un risque aussi bien pour l´équipe soignante que pour les patients. Le type d´amalgame utilisé joue un rôle important dans le degré d´exposition. L´utilisation de l´amalgame en vrac multiplie les moments d´expositions potentielles depuis le remplissage de la cuve à mercure de l´amalgamateur avec le risque accru de perte, les opérations d´entretien et lors de la récupération de l´amalgame malaxé. Il s´agit d´une technique hautement polluante. Les utilisateurs des capsules ne sont potentiellement exposés qu´à l´ouverture de ces dernières [31]. L´entretien des locaux par aspiration ou par simple balayage à sec peut remettre en suspension dans l´air de fines particules déposées au sol et augmenter ainsi l´exposition [4]. Un lavage classique à l´eau semble plus protecteur. La dépose et le polissage des amalgames est une manœuvre génératrice de vapeur surtout lorsque le travail est effectué à sec et sans jet d´eau de refroidissement [4, 15]. Le risque environnemental est en rapport avec la gestion des déchets. Dans notre cas, 71,43% utilisent le réseau municipal responsable de la collecte des déchets ménagers. Dans une enquête réalisée dans la région de Rabat, capitale du Maroc, ce taux est de 58% en 2016 [10] alors qu´il était de 69,5% en 2011 [11].

**Problèmes de santé:** les dysfonctionnements neuro-psychologiques sont la première préoccupation sanitaire chez 46,35% des participants. Bien que cette proportion semble être importante, elle reste comparable aux taux retrouvés dans d´autres pays. Dans une étude anglaise [32], 25,53% des dentistes souffrent d´épuisement émotionnel, 34,42% de réduction d´accomplissement personnel et 8,88% de dépersonnalisation. En Espagne [33], les proportions sont plus importantes avec respectivement 55,6%, 6,9% et 54,3%. Une étude récente réalisée à Hong Kong [34] révéla des taux comparables des mêmes troubles (respectivement 25,4%, 39,0% et 17,2%). Les auteurs de ces différentes études ont cherché des corrélations entre ces troubles et le stress professionnel qui mène au *burnout* et à la longue à la dépression [35]. La piste du *burnout* est certes plausible mais n´est pas unique. Aucune corrélation avec le mercure dentaire n´a été recherchée dans ces études. Une étude publiée en 2008 sur les problèmes de santé auto-déclarés par les médecins dentistes en Lituanie [36] avait révélé la présence de fortes proportion de fatigue (91,5%), de maux de tête (83,6%) et de douleurs thoraciques (38,4%). Ces troubles sont ressentis de façon chronique par les praticiens. Un modèle de régression logistique a montré un lien significatif entre l´âge et l´état de santé. Chaque année d´âge, et par conséquent d´ancienneté d´exercice, a un impact négatif sur la santé des médecins dentistes et diminue de 6% la probabilité d´être en bonne ou très bonne santé. La présence de dérèglements psychologiques et notamment l´anxiété, la dépression et la fatigue chronique avec une exposition chronique au mercure peut faire suspecter un microhydrargyrisme [37]. L´intensité de l´exposition au mercure en rapport avec l´application des mesures de protection et d´hygiène peut expliquer la différence constatée dans la répartition de certains troubles neuropsychologiques chez les dentistes [38]. Une concentration atmosphérique de mercure qui dépasse le seuil de 30 μg/m^3^ peut provoquer des signes neurologiques tels que la diminution des capacités psychomotrices, des tremblements objectivement détectables et des désordres dans la conduction des nerfs périphériques. Des symptômes subjectifs comme la fatigue, l´irritabilité et l´anxiété etc., peuvent apparaitre également à partir de ce seuil [31]. Dans plusieurs études [4], la limite de 30 μg/m^3^ est largement dépassée dans des proportions non négligeables de cabinets dentaires. Dans notre contexte, malgré l´absence de mesures atmosphériques objectivement chiffrées, la présence concomitante de plusieurs facteurs favorisant la pollution de l´air ambiant et l´absence de décontamination des locaux laisse présager la découverte de taux atmosphériques conséquents en cas de mesure. Les troubles dermatologiques sont assez fréquents et le taux retrouvé est comparable avec d´autres études [39]. Les dermatoses allergiques de contact avec les différents produits utilisés en dentisterie sont souvent avancées comme étiologie mais des études ont retrouvé un lien significatif entre les signes dermatologiques et l´exposition au mercure [40]. Les proportions des autres troubles sont relativement faibles mais les signes d´une intoxication chronique au mercure peuvent être infracliniques et une démarche diagnostique exploratrice est nécessaire dans le cas d´une étude étiologique qui s´avère parfaitement justifiée au vu des résultats retrouvés.

## Conclusion

L´exposition au mercure chez les médecins dentistes des régions étudiées est réelle surtout pour les utilisateurs des amalgames dentaires. En l´absence de décontamination, l´exposition persiste même après l´arrêt ou la réduction drastique de son utilisation. Plusieurs conditions liées à l´environnement de travail ou aux habitudes professionnelles nécessitent une attention particulière et des mesures de prévention efficaces pour une meilleure protection de l´équipe soignante et de l´environnement. Les plaintes sanitaires rapportées par notre population pourraient être associées, du moins en partie, à l´exposition chronique au mercure, d´où la nécessité d´une enquête épidémiologique à visée étiologique avec le mercure comme facteur de risque pour confirmer ou infirmer cette hypothèse.

### Etat des connaissances sur le sujet

- La toxicité du mercure est un fait mondialement admis (convention de Minamata);
- Les médecins dentistes qui utilisent les amalgames dentaires dans leur pratique sont exposés au mercure à des degrés variables
- Les effets de cette exposition sur la santé des praticiens sont encore sujets de controverses; l´impact du mercure sur la santé dépend de l´intensité de l´exposition mais aussi de sa durée.

### Contribution de notre étude à la connaissance

- Aperçu sur la situation au Maroc concernant l´exposition des médecins dentistes au mercure et ce vu l´absence de données épidémiologiques sur le sujet;
- Mise en évidence des facteurs qui augmentent l´intensité de l´exposition à ce facteur de risque ce qui permet de mieux cibler les mesures de sensibilisation et de prévention;
- Identifier les doléances sanitaires de la population étudiée pour juger de la pertinence d´une étude étiologique ultérieure.
